# Papillary Thyroid Microcarcinoma: Diagnostic and Histopathological Insights From a Case Report

**DOI:** 10.7759/cureus.106481

**Published:** 2026-04-05

**Authors:** Sanjana Malhotra, Mohd Nazar Rana, Yuthika Yadav, Girik Subudhi, Mohammad Arif

**Affiliations:** 1 Oral Pathology and Microbiology, Subharti Dental College and Hospital, Meerut, IND; 2 Periodontology, Subharti Dental College and Hospital, Meerut, IND; 3 General Practice, Subharti Medical College and Chhatrapati Shivaji Subharti (CSS) Hospital, Meerut, IND; 4 General Medicine, Rohilkhand Medical College and Hospital, Bareilly, IND; 5 Surgery, Subharti Medical College and Chhatrapati Shivaji Subharti (CSS) Hospital, Meerut, IND

**Keywords:** hürthle cell neoplasm, papillary thyroid microcarcinoma, parathyroid adenoma, primary hyperparathyroidism, thyroid nodule

## Abstract

The coexistence of primary hyperparathyroidism and thyroid nodular disease is frequently encountered in clinical practice, and the simultaneous presence of primary hyperparathyroidism and papillary thyroid microcarcinoma is clinically significant and may pose diagnostic and therapeutic challenges. A 63-year-old postmenopausal woman presented with a short history of painless left-sided neck swelling. Initial ultrasonography revealed a bulky left thyroid lobe with a dominant isoechoic nodule, and fine-needle aspiration cytology (FNAC) categorised the lesion as Bethesda Category IV, suspicious for a Hürthle cell neoplasm. Biochemical evaluation demonstrated markedly elevated intact parathyroid hormone levels, consistent with primary hyperparathyroidism. Subsequent parathyroid scintigraphy localised left-sided parathyroid adenomatous disease. The patient underwent left thyroid lobectomy with excision of the suspected parathyroid tissue. Histopathological examination incidentally revealed a 3 mm papillary thyroid microcarcinoma, with no evidence of lymphovascular invasion or extrathyroidal extension and with clear surgical margins. This case underscores the importance of comprehensive preoperative evaluation in patients with concomitant thyroid and parathyroid abnormalities. Early recognition of synchronous pathology allows for appropriate surgical planning, reduces the need for reoperation, and contributes to favourable clinical outcomes. Reporting such cases enhances awareness of this association and supports an integrated approach to the management of complex endocrine neck disorders.

## Introduction

Primary hyperparathyroidism and thyroid nodular disease are conditions that often go hand in hand because of their embryological derivation, anatomical proximity, and close overlap in diagnosis and surgical management [1]. This association has become increasingly apparent with the widespread use of high-resolution neck ultrasonography, biochemical screening, and functional nuclear imaging, which have led to an increased number of incidental findings involving both thyroid and parathyroid abnormalities [2,3]. As a result, clinicians are increasingly challenged by the need to evaluate and manage coexisting endocrine lesions in the neck [4].

Thyroid nodules are common among patients with primary hyperparathyroidism, and the occurrence of differentiated thyroid carcinoma in this context has significant clinical implications [5]. A papillary thyroid tumour measuring less than 1 cm is known as papillary thyroid microcarcinoma and is generally indolent in nature; it is often discovered incidentally during histopathological examination following thyroidectomy performed for a benign or indeterminate lesion [6,7]. Although it generally has a favourable prognosis, failure to detect concurrent thyroid malignancy preoperatively may affect surgical planning and increase the likelihood of reoperation [8].

Primary hyperparathyroidism, in turn, is a hormonally active disease that may present with systemic manifestations, including renal and skeletal complications, and may require definitive surgical treatment [9]. When both conditions coexist, preoperative assessment must be more thorough in order to reach an accurate diagnosis and ensure optimal treatment [10]. Such cases have been reported in the literature and have highlighted the importance of combining diagnostic modalities and interdisciplinary collaboration in the management of complex endocrine disorders involving the thyroid and parathyroid glands.

## Case presentation

Patient information and clinical history

A 63-year-old postmenopausal woman presented with a slowly enlarging swelling on the left side of the neck, which she had noticed for 15-20 days before examination. It was painless and was not accompanied by fever, dysphagia, hoarseness of voice, or compressive symptoms. The patient was a known case of systemic hypertension (HTN) and chronic kidney disease and had previous biochemical results suggestive of hypercalcaemia. She denied any family history of thyroid or parathyroid disease. Physical examination revealed a hard, non-tender mass in the left side of the neck that moved with deglutition. There was no visible cervical lymphadenopathy.

Neck USG findings

**Figure 1:**
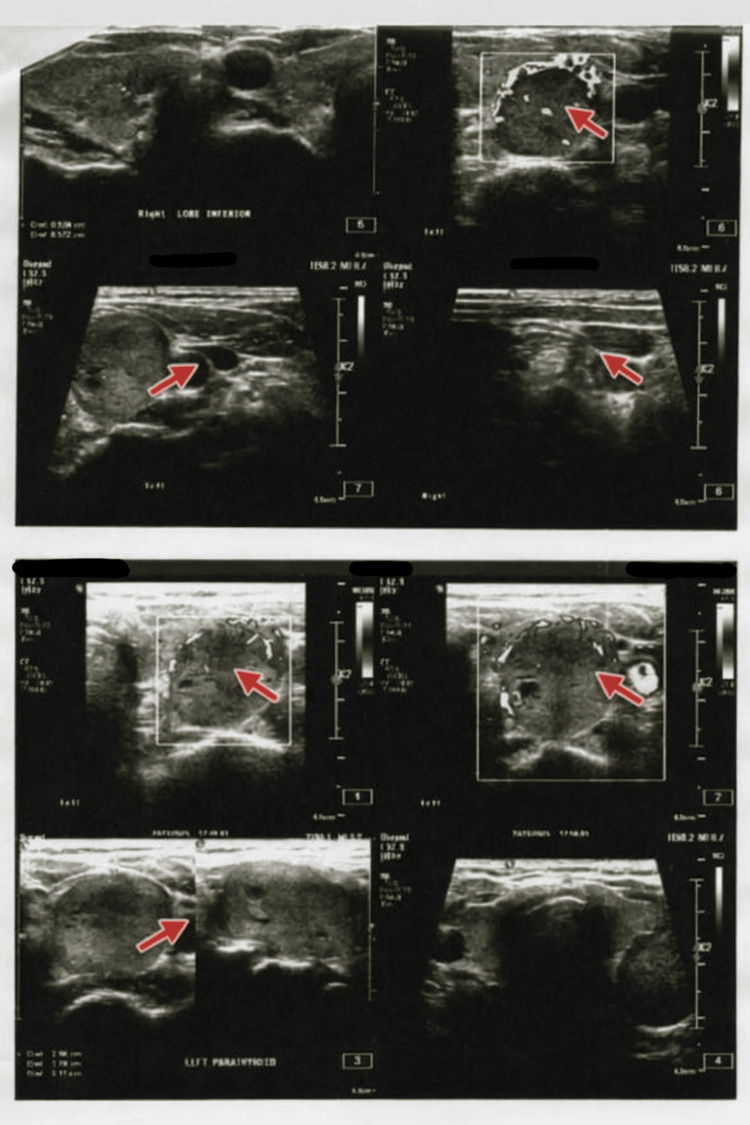
Ultrasound images of the left thyroid nodule.

Ultrasonic images of the neck showed that the right thyroid lobe was normal in size and structure, with intact echotexture and no lesions. The left thyroid lobe was enlarged and bulky and contained a well-defined nodule. The left lobe measured approximately 31 × 23 × 16 mm, and the nodule measured 26 × 24 × 15 mm. No cystic degeneration or calcifications were detected. The thyroid isthmus was normal in thickness, and the neck vessels and muscles were unremarkable. There was no cervical lymphadenopathy. Based on these findings, further evaluation with fine-needle aspiration cytology (FNAC) was recommended. Figure 1 shows enlargement of the left thyroid lobe containing a distinct isoechoic nodular lesion on neck USG.

FNAC

**Figure 2:**
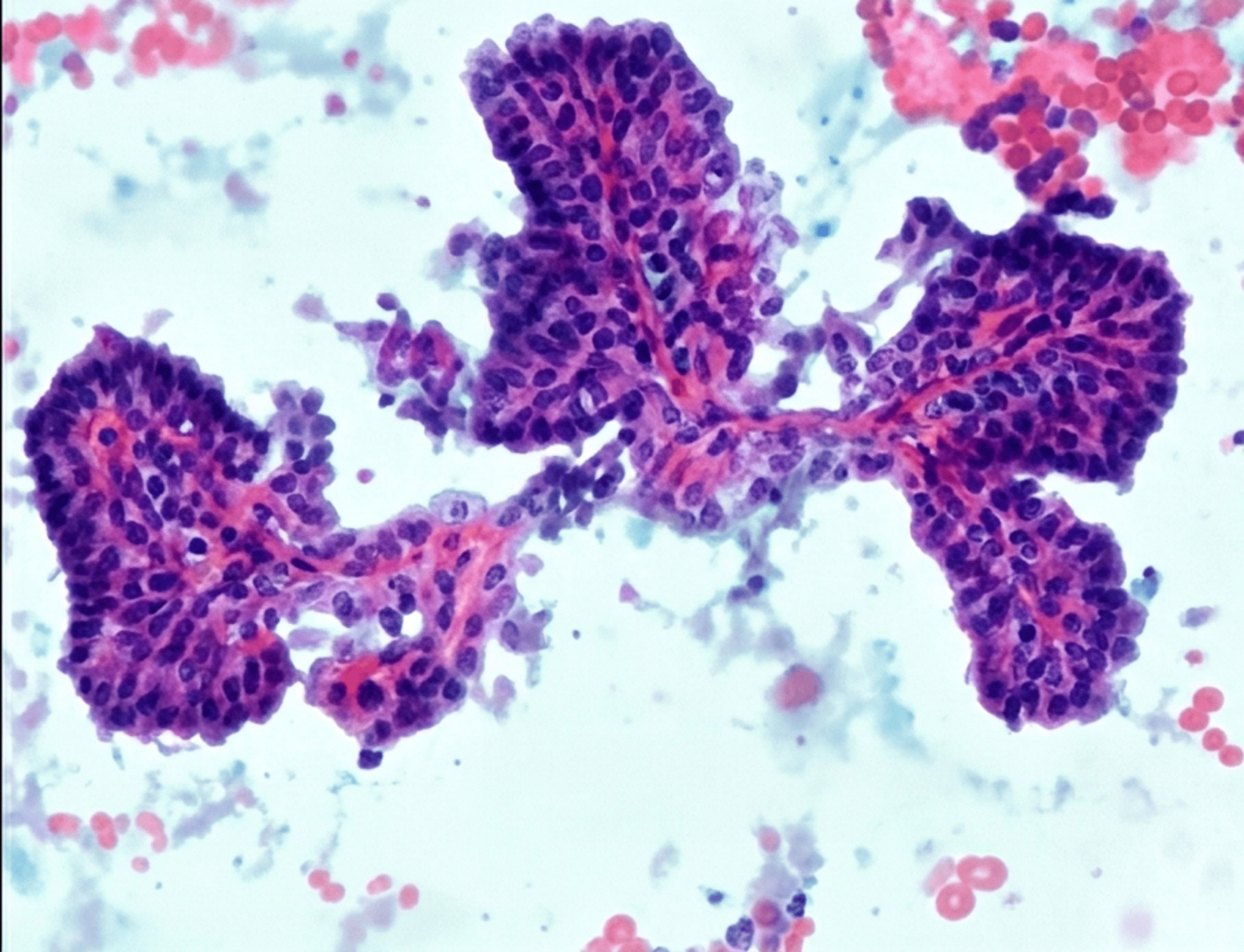
Fine-needle aspiration cytology (FNAC) of the left thyroid nodule. FNAC smear from the left thyroid nodule showing high cellularity, with sheets and clusters of Hürthle cells dispersed in a haemorrhagic background (H&E stain, ×400). The cells exhibit abundant granular eosinophilic cytoplasm and round, centrally placed nuclei with prominent nucleoli. Occasional bare nuclei are also noted. These cytomorphological features are suggestive of a Hürthle cell-predominant lesion and were categorised as Bethesda Category IV - Suspicious for Hürthle cell neoplasm.

FNAC was performed under ultrasound guidance from the left thyroid nodule. Cytological analysis revealed cellular smears in a haemorrhagic background, with sheets, clusters, and singly scattered Hürthle cells. Some bare nuclei were also reported. The lesion was classified under the Bethesda System for Reporting Thyroid Cytopathology as Bethesda Category IV - suspicious for Hürthle cell neoplasm. Since the malignant potential of this category is indeterminate, surgical excision with histopathological correlation was suggested (Figure 2).

Biochemical and hormonal evaluation

Additional laboratory testing showed a markedly elevated intact parathyroid hormone (PTH) level of 729.9 pg/mL (reference range: 15-65 pg/mL), indicating severe hyperparathyroidism. These results, together with the patient’s known hypercalcaemia and renal disease, supported a diagnosis of primary hyperparathyroidism. As a thyroid nodule and biochemical hyperparathyroidism coexisted, functional imaging was requested to localise a possible source of parathyroid tissue. Biochemical analysis therefore demonstrated a considerably increased intact PTH level, supporting the diagnosis of primary hyperparathyroidism.

Parathyroid scintigraphy

Dual-phase parathyroid scintigraphy was performed using Technetium-99m sestamibi and pertechnetate subtraction. Early images showed physiological tracer uptake in the right thyroid lobe and salivary glands. Delayed images revealed persistent tracer retention in the upper and lower poles of the left thyroid region, consistent with probable left superior and inferior parathyroid adenomas. The upper pole of the left thyroid lobe also showed an incidental cold nodule, which required further clinicopathological correlation. Scintigraphy thus demonstrated delayed radiotracer retention in the left parathyroid region on sestamibi imaging, indicative of a parathyroid adenoma.

Systemic imaging evaluation

Systemic involvement and complications of hyperparathyroidism were assessed by whole-abdomen USG. Mild fatty infiltration of the liver (Grade I) was detected. Both kidneys were normal in size and position, but focal cortical scarring was observed in the right kidney, and mildly increased cortical echogenicity with a small cortical cyst was observed in the left kidney. No adrenal masses, ascites, or significant pelvic pathology were observed.

Surgical management and gross examination

Surgical intervention was undertaken in view of the presence of primary hyperparathyroidism and a suspicious thyroid nodule. Left thyroid lobectomy and excision of the suspected parathyroid tissue were performed. Gross examination revealed a nodular tissue specimen measuring approximately 3 × 2 × 1 cm. On serial sectioning, a small greyish-white focus measuring about 0.3 cm was identified in the thyroid tissue. The resected specimen was then subjected to definitive histopathological examination.

Histopathological findings

**Figure 3:**
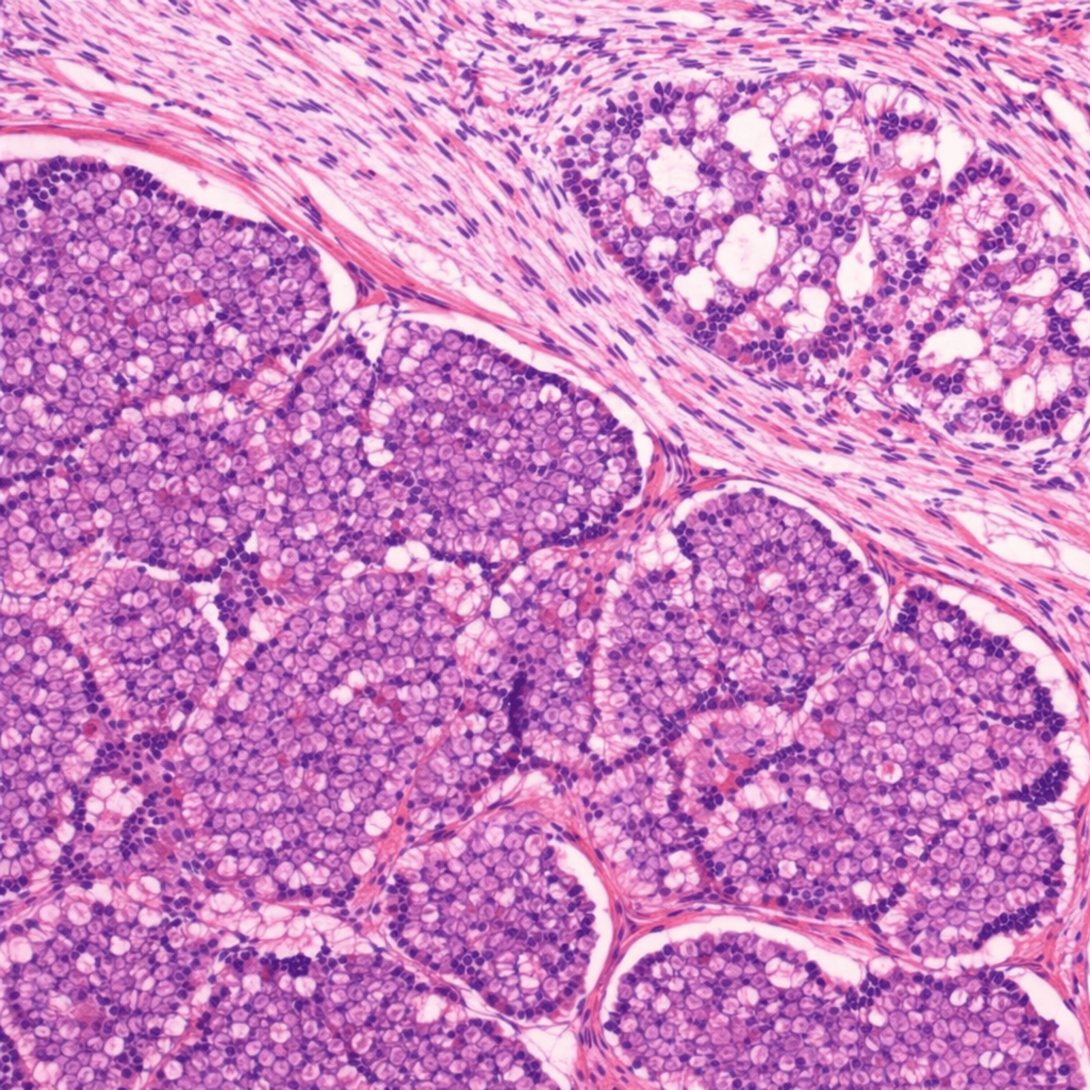
Histopathological examination of resected thyroid tissue. Photomicrograph of a H&E-stained section from the resected left thyroid lobe showing a well-circumscribed microscopic tumour focus measuring approximately 3 mm (×400). The lesion demonstrates follicular-patterned cells with classical nuclear features of papillary thyroid carcinoma, including nuclear enlargement, overlapping, optically clear chromatin, nuclear grooves, and occasional intranuclear pseudoinclusions. No evidence of lymphovascular invasion or extrathyroidal extension is identified. Surgical margins are free of tumour, consistent with papillary thyroid microcarcinoma.

Microscopic examination revealed a well-circumscribed tumour focus measuring about 3 mm, composed of follicular-patterned cells with classical nuclear features of papillary thyroid carcinoma, including nuclear enlargement, overlapping, clear chromatin, nuclear grooves, and pseudoinclusions (Figure 3). No psammoma bodies were identified. There was no evidence of vascular invasion, lymphatic invasion, or extrathyroidal extension. The surgical margins were clear. The final diagnosis was papillary thyroid microcarcinoma (follicular architecture) of the left thyroid, arising in the background of primary hyperparathyroidism. Microscopic examination confirmed features consistent with papillary thyroid microcarcinoma in the absence of adverse pathological parameters.

## Discussion

This case highlights a complex presentation of a Hürthle cell neoplasm in a 63-year-old postmenopausal female, characterized by significant biochemical, radiological, and cytological correlations. The patient demonstrated marked hypercalcemia (serum calcium 13.2 mg/dL) with a profoundly elevated intact PTH level (729.9 pg/mL), strongly suggestive of a PTH-dependent hypercalcemic state, which clinically manifested as acute kidney disease (AKD) and necessitated nephrology evaluation and active metabolic management. USG of the neck (Figure 1) revealed a bulky left thyroid lobe harboring a large, well-defined isoechoic nodule measuring approximately 26 × 24 × 15 mm, while the right lobe and isthmus were unremarkable, and no cervical lymphadenopathy was identified, indicating a localized thyroid pathology. Subsequent ultrasound-guided FNAC (Figure 2), reported under the Bethesda System, categorized the lesion as Category IV, suspicious for Hürthle cell neoplasm, based on the presence of sheets and clusters of oncocytic cells with abundant granular cytoplasm, supporting a neoplastic rather than inflammatory process. Whole-abdomen USG further demonstrated systemic effects of prolonged metabolic imbalance, including grade I fatty liver changes, focal cortical scarring of the right kidney, mild cortical echogenicity of the left kidney with a small cortical cyst, and absence of obstructive uropathy, collectively reinforcing the diagnosis of hypercalcemia-related renal involvement rather than primary renal pathology. The coexistence of severe hypercalcemia, elevated PTH levels, thyroid neoplasm with oncocytic cytology, and renal dysfunction underscores the importance of comprehensive evaluation to distinguish primary thyroid pathology from possible parathyroid-related endocrine dysfunction or dual pathology. This case emphasizes that Hürthle cell neoplasms, though primarily thyroid-derived, may present with significant systemic metabolic derangements, necessitating a multidisciplinary diagnostic approach and careful clinicoradiological-pathological correlation to guide definitive surgical and therapeutic management.

Furthermore, functional imaging with PET demonstrated a metabolically active FDG-avid lesion localized to the left thyroid lobe, corresponding to the previously identified nodule, with no evidence of distant metastatic disease.

Histopathological examination (Figure 3) of multiple sections from the left thyroid lobe revealed a small focus of tumor measuring 3 mm, composed of tumor cells arranged predominantly in a follicular pattern, with some follicles containing thick colloid. The tumor cells exhibited characteristic nuclear features, including nuclear enlargement, chromatin clearing, nuclear grooves, and intranuclear pseudoinclusions. Psammoma bodies were not identified. The tumor was located 1 mm from the surgical excision margin. Focal lymphocytic infiltration was noted in the surrounding thyroid tissue.

These findings, together with the histopathological examination, supported the final diagnosis of papillary thyroid microcarcinoma. The absence of extra-thyroidal uptake further aided in staging and reinforced the suitability of definitive surgical management.

Primary hyperparathyroidism and thyroid nodular disease have been documented to coexist, with a reported prevalence of 20-60 per cent, predominantly due to their shared embryological origin and anatomical proximity, as well as the increased use of high-resolution neck imaging [11]. The clinical significance of this relationship lies in its potential impact on surgical decision-making and patient outcomes. Incidentally, papillary thyroid microcarcinoma is often identified on histopathological examination after thyroid surgery undertaken for benign or indeterminate thyroid lesions [12].

In the current case, the patient presented with biochemical evidence of primary hyperparathyroidism, confirmed by elevated PTH levels and functional imaging suggestive of parathyroid adenomatous disease. At the same time, the presence of a thyroid nodule categorised as Bethesda Category IV made surgical exploration equally significant, highlighting the importance of comprehensive preoperative evaluation in the presence of both cytological and biochemical abnormalities. Indeterminate thyroid cytology, particularly in Hürthle cell-predominant lesions, poses a diagnostic challenge because it encompasses both benign and malignant possibilities and, in most cases, requires definitive histopathological evaluation [13].

Surgically, the ability to identify both thyroid and parathyroid pathology at the same time allows all procedures to be performed in a single operation, which helps to mitigate the possibility of missed diagnosis and prevents postoperative morbidity caused by reoperation [14]. The indolent nature of papillary thyroid microcarcinoma is reflected in the incidental discovery of the lesion in this case, which did not show any adverse pathological features and carries an excellent prognosis with appropriate management [15]. This case justifies the importance of a multidisciplinary approach and comprehensive diagnostics in the treatment of complex endocrine neck disorders.

Building on these observations, this case also highlights the nuanced decision-making required when managing coexisting endocrine pathologies of the neck. The overlap of clinical, biochemical, and radiological findings between thyroid and parathyroid disorders often complicates preoperative planning, particularly when cytology yields indeterminate results. In such scenarios, reliance on a single diagnostic modality may be insufficient; rather, an integrated assessment incorporating biochemical markers, high-resolution imaging, cytopathology, and clinical judgment becomes essential. The coexistence of primary hyperparathyroidism with a Bethesda Category IV thyroid nodule underscores the need for heightened vigilance, as the presence of one endocrine pathology does not exclude the possibility of another. Careful interpretation of preoperative findings enables tailored surgical strategies that address all suspected lesions while minimizing operative risk and preserving critical structures in the neck.

Furthermore, the incidental identification of papillary thyroid microcarcinoma in this patient reinforces the evolving understanding of thyroid malignancies detected during surgery for other indications. While such microcarcinomas generally demonstrate indolent biological behaviour and favourable outcomes, their recognition carries implications for postoperative surveillance and long-term management. Early detection, even when incidental, allows for appropriate risk stratification and individualized follow-up protocols, thereby reducing the likelihood of disease progression or recurrence. Overall, this case exemplifies the value of comprehensive evaluation and coordinated care among endocrinologists, radiologists, pathologists, and surgeons, ensuring optimal outcomes in patients with complex and synchronous endocrine neck diseases.

## Conclusions

The comorbidity of primary hyperparathyroidism and papillary thyroid microcarcinoma, as observed in this case, is clinically pertinent and highlights the need for detailed evaluation of patients presenting with cervical masses and endocrine dysfunction. The combination of laboratory tests, high-resolution imaging, and cytological examination strongly indicates that close preoperative assessment is required in cases of concurrent biochemical hyperparathyroidism and an indeterminate thyroid nodule. Synchronous thyroid and parathyroid pathology should be identified whenever possible, as both can be addressed in a single operation; thus, patient morbidity can be minimized and the likelihood of reoperation reduced. The incidental discovery of papillary thyroid microcarcinoma on histopathological analysis further supports the generally indolent nature of this malignancy and its favourable prognosis, provided it is managed appropriately. The need for interdisciplinary collaboration among endocrinologists, surgeons, radiologists, and pathologists in this case illustrates that the most accurate diagnoses and the most effective treatment can be achieved through such an approach. Reporting such cases contributes to a deeper understanding of the complex interrelationship between thyroid and parathyroid diseases and underscores the importance of integrated treatment strategies in endocrine surgery.
